# Efficacy of the Enquiring About Tolerance (EAT) study among infants at high risk of developing food allergy

**DOI:** 10.1016/j.jaci.2019.06.045

**Published:** 2019-12

**Authors:** Michael R. Perkin, Kirsty Logan, Henry T. Bahnson, Tom Marrs, Suzana Radulovic, Joanna Craven, Carsten Flohr, E.N. Mills, Serge A. Versteeg, Ronald van Ree, Gideon Lack, Enquiring About Tolerance (EAT) study team, Louise Young, Victoria Offord, Mary DeSousa, Jason Cullen, Katherine Taylor, Anna Tseng, Bunmi Raji, Sarah Byrom, Gillian Regis, Charlie Bigwood, Charlotte Stedman, Sharon Tonner, Emily Banks, Yasmin Kahnum, Rachel Babic, Ben Stockwell, Erin Thompson, Lorna Wheatley, Devi Patkunam, Kerry Richards, Ewa Pietraszewicz, Alick Stephens, Asha Sudra, Victor Turcanu

**Affiliations:** a Population Health Research Institute, St George's, University of London, London, United Kingdom; b Paediatric Allergy Research Group, Department of Women and Children's Health, School of Life Course Sciences, King's College London, London, United Kingdom; c Benaroya Research Institute, Seattle, Wash; d Unit for Population-Based Dermatology Research, St John's Institute of Dermatology, School of Basic and Medical Biosciences, Faculty of Life Sciences & Medicine, King's College, London, United Kingdom; e School of Biological Sciences, Division of Infection, Immunity and Respiratory Medicine, Manchester Academic Health Science Centre, Manchester Institute of Biotechnology, University of Manchester, Manchester, United Kingdom; f Department of Experimental Immunology, Academic Medical Center, Amsterdam, The Netherlands; g Department of Otorhinolaryngology, Academic Medical Center, Amsterdam, The Netherlands

**Keywords:** Food allergy, diet, allergens, infancy, breastfeeding, randomized controlled trial, adherence, EAT, Enquiring About Tolerance, EIG, Early introduction group, LEAP, Learning Early About Peanut, OR, Odds ratio, SCORAD, Scoring Atopic Dermatitis, SIG, Standard introduction group

## Abstract

**Background:**

The Enquiring About Tolerance (EAT) study was a randomized trial of the early introduction of allergenic solids into the infant diet from 3 months of age. The intervention effect did not reach statistical significance in the intention-to-treat analysis of the primary outcome.

**Objective:**

We sought to determine whether infants at high risk of developing a food allergy benefited from early introduction.

**Methods:**

A secondary intention-to-treat analysis was performed of 3 groups: nonwhite infants; infants with visible eczema at enrollment, with severity determined by SCORAD; and infants with enrollment food sensitization (specific IgE ≥0.1 kU/L).

**Results:**

Among infants with sensitization to 1 or more foods at enrollment (≥0.1 kU/L), early introduction group (EIG) infants developed significantly less food allergy to 1 or more foods than standard introduction group (SIG) infants (SIG, 34.2%; EIG, 19.2%; *P* = .03), and among infants with sensitization to egg at enrollment, EIG infants developed less egg allergy (SIG, 48.6%; EIG, 20.0%; *P* = .01). Similarly, among infants with moderate SCORAD (15-<40) at enrollment, EIG infants developed significantly less food allergy to 1 or more foods (SIG, 46.7%; EIG, 22.6%; *P* = .048) and less egg allergy (SIG, 43.3%; EIG, 16.1%; *P* = .02).

**Conclusion:**

Early introduction was effective in preventing the development of food allergy in specific groups of infants at high risk of developing food allergy: those sensitized to egg or to any food at enrollment and those with eczema of increasing severity at enrollment. This efficacy occurred despite low adherence to the early introduction regimen. This has significant implications for the new national infant feeding recommendations that are emerging around the world.

## Graphical abstract

**Figure:**
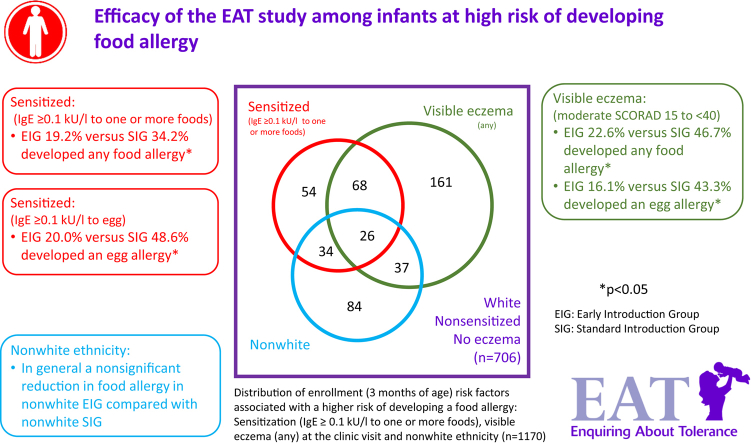

The Enquiring About Tolerance (EAT) Study was conceived to reduce the burden of food allergies through early oral tolerance induction to specific food antigens.^1^ Rates of adherence to the challenging early introduction protocol were low, with 43% of adherence-evaluable EIG infants (34% of all EIG infants) consuming the per-protocol consumption threshold of recommended allergenic foods. This level of adherence was associated with a nonsignificant reduction in IgE-mediated food allergy to 1 or more of the 6 intervention foods.^2^

At a food-specific level, adherence varied, with some foods being easier to consume than others: egg at 43.1% and peanut at 61.9%.^1^ In the intention-to-treat analysis both foods showed nonsignificant reductions in allergy prevalence of 31% and 51% for egg and peanut, respectively.^2^ Per-protocol effects were significantly stronger and in keeping with Learning Early About Peanut (LEAP) allergy study effect estimates.^3^ However, per-protocol analyses are susceptible to bias depending on what factors determined adherence in the study.

Well-recognized factors that render an infant from the general population to be at greater risk of a food allergy include nonwhite ethnicity,^4^ early-onset eczema,^5^ and early-onset sensitization to a food.^6^, ^7^ In the EAT study we have previously confirmed that nonwhite ethnicity (odds ratio [OR], 2.09; 95% CI, 1.19-3.66; *P* = .01) and enrollment visible eczema (3 months of age; OR, 6.09; 95% CI, 3.67-10.1; *P* < .001) were both significantly associated with developing a food allergy.^2^

At the time of publication of the EAT study's principal results, the enrollment sensitization data available were the skin prick test results, which took place, by design, only in the early introduction group (EIG). Enrollment skin prick test sensitization (any positive wheal response to ≥1 foods) was significantly associated with having a food allergy in the EIG (OR, 5.71; 95% CI, 1.99-16.3; *P* = .001).

The absence of comparator data from the standard introduction group (SIG) precluded comparisons between the 2 groups in terms of efficacy of the EAT intervention in infants sensitized at enrollment. Following publication of the primary EAT study findings, we secured funding to process IgE results for this large sample set. Infants were defined as sensitized at enrollment if they had specific IgE antibodies present to 1 or more of the 6 allergenic foods at enrollment at 3 months of age.

We now present the findings of the efficacy of the EAT study in these groups of infants at high risk of developing a food allergy. The efficacy analysis of the subgroup of infants with eczema at enrollment was stipulated in the EAT study protocol *a priori*. The efficacy of the EAT study intervention in nonwhite participants and those sensitized at enrollment are secondary analyses.

## Methods

### Participants

**Fig E1:**
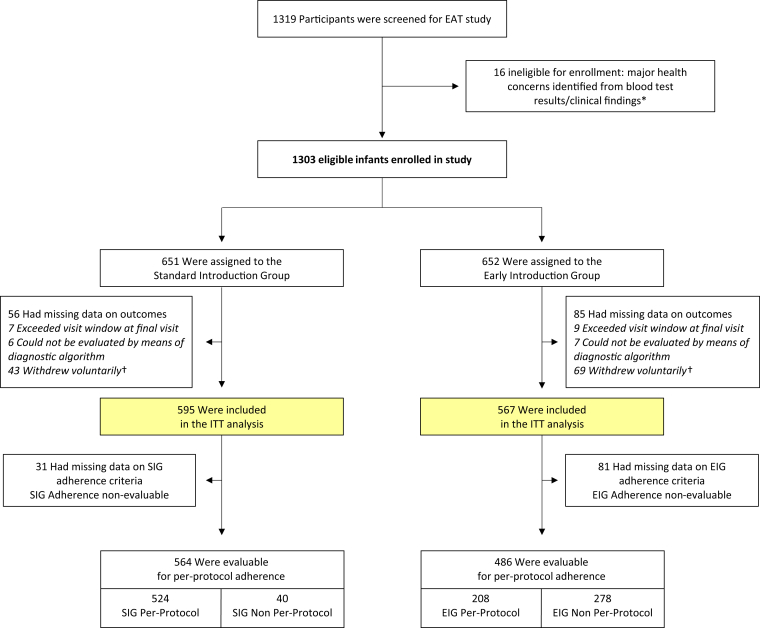
EAT enrollment and randomization. Baseline visits occurred when participants were 3 months of age. *Eight infants randomized to each group were found to have significant health issues either on blood testing or on clinical examination at the enrollment visit, rendering them ineligible for enrollment: conditions included severe vitamin D deficiency, severe iron deficiency, severe failure to thrive, familial hypercholesterolemia, congenital stridor, epidermolysis bullosa, and cartilage-hair hypoplasia syndrome. †Forty-three participants in the SIG and 69 participants in the EIG withdrew voluntarily from the study. Reasons given were as follows: concerns about blood tests (SIG, 0; EIG, 2), emigration (SIG, 10; EIG, 12), expenses (SIG, 1; EIG, 1), family health issues (SIG, 3; EIG, 0), family issues (SIG, 2; EIG, 4), no reason given (SIG, 11; EIG, 16), lost contact with family (SIG, 15; EIG, 28), too far to travel for study assessments (SIG, 0; EIG, 1), and unhappy participating in the study (SIG, 1; EIG, 5).

One thousand three hundred and three 3-month-old infants were recruited from the general population in England and Wales through direct advertising and enrolled between November 2, 2009, and July 30, 2012. The CONSORT figure for the EAT study is shown in Fig E1 in this article's Online Repository at www.jacionline.org. Full methodological details, the trial protocol, and the statistical analysis plan (original documents and final versions, with summary of all changes made to both documents) and other CONSORT information for the primary outcome of the EAT study are published elsewhere.^1^, ^2^ The trial was registered with the ISRCTN (registration no. 14254740). All participants were healthy, exclusively breastfed, and born at term (≥37 weeks' gestation). Ethnic origin of the child was based on self-defined parental ethnicity coded by the classification used in the 2001 UK Census.^8^ Ethical approval for the EAT study was provided by St Thomas' Hospital REC (REC reference 08/H0802/93), and informed consent was obtained from the parents of all children enrolled in the study.

### EAT study intervention

In brief, participants were randomized to the SIG or EIG. The SIG was asked to exclusively breastfeed to around 6 months of age. Allergenic food introduction beyond this point was at parental discretion. EIG infants continued to breastfeed while sequentially introducing 6 allergenic foods: cow's milk yogurt; then peanut, hard-boiled egg, sesame, and whitefish (cod) in a random order; and finally wheat. By week 6, EIG infants were ideally consuming the required amount of all 6 allergenic foods each week.

### Eczema assessment

Scheduled clinic visits took place at enrollment and 1 and 3 years of age. All children were examined for eczema at all 3 clinic visits using the UK Diagnostic Criteria–based photographic protocol of the International Study of Asthma and Allergies in Childhood Phase Two.^9^ Disease severity was determined by using the Scoring Atopic Dermatitis (SCORAD) index.^10^ SCORAD was categorized as mild (<15), moderate (15 to <40), and severe (≥40).^10^

### Sensitization

Only infants in the EIG underwent skin prick tests at enrollment. SIG participants did not undergo skin prick tests at enrollment because this would likely have influenced a family's decision on when to introduce allergenic foods into their child's diet. The desire was for SIG participants to introduce allergenic foods at the same time that they would have done if they had not been participating in the study.

In contrast, blood sampling was attempted on all infants at enrollment. Samples were then stored until completion of the study. IgE levels to each of the 6 individual foods were then measured with the ImmunoCAP assay system (Phadia, Uppsala, Sweden).

### Primary outcome

All children with a positive skin prick test response to 1 or more of the 6 intervention foods at the 1-year and/or 3-year assessments or a history of a positive challenge at less than 1 year of age were considered for a food challenge. The decision to challenge and the timing and type of challenge undertaken were based on the participant's study group and frequency of consumption status.^2^ In the EAT study the primary outcome was allergy proven by double-blind challenge where possible, to 1 or more of the 6 early introduction foods. One thousand one hundred seventy-three (90.0%) of the participants attended the final 3-year visit within the scheduled visit window (by 4 years of age), and the primary outcome could be determined in 1178 (90.4%) participants. Although the study was not specifically powered to look at individual food allergy outcomes, there were sufficient cases of peanut and egg allergy to analyze these outcomes. There were insufficient cases of allergy to any of the other 4 foods (sesame, wheat, cow's milk, and fish) to undertake subgroup analyses for these foods.

Diagnostic cut-off values for predicting food allergy based on skin prick test results have been shown to have lower thresholds in younger children.^11^ Safety was paramount in the EAT study and, given that the presence of a positive skin prick test response at enrollment resulted in a food challenge being undertaken before home consumption was allowed, a wheal of any size was deemed positive to minimize false-negative test results. Although IgE sensitization data are presented by using a standard threshold of 0.35 kU/L or greater, given the very young age, a lower threshold of 0.1 kU/L or greater was used for the primary analyses of food allergy outcomes. For the outcome of food allergy to 1 or more of the 6 early introduction foods, sensitization at enrollment was defined as sensitization of 0.1 kU/L or greater to any 1 of the 6 foods. For peanut and egg allergy outcomes, sensitization at enrollment refers to sensitization of 0.1 kU/L or greater to the specific food.

### Statistical analyses

Chi-squared tests or Fisher exact tests (where appropriate) were undertaken for unadjusted analyses. A formal test for interaction was undertaken in a logistical model, including study group and sensitization to 1 or more foods (for the outcome of allergy to any food) or sensitization to a specific food (for the outcome of food allergy to that food). An interaction term was included between the study group and sensitization variables. Analyses were undertaken with Stata 15 software (StataCorp, College Station, Tex). The EAT data set (ITN900AD) is available through TrialShare, a public Web site managed by the Immune Tolerance Network (www.itntrialshare.org).

## Results

Infants were enrolled in the EAT study at 3 months of age (mean age at enrollment, 14.7 weeks).

### Eczema

Visible eczema was present at the enrollment visit in 24.3% (317/1302) of participants. Of the 317 infants with eczema, most was mild: mild SCORAD (<15) in 76.3% (n = 242), moderate SCORAD (15 to <40) in 20.8% (n = 66), and severe SCORAD (≥40) in 2.8% (n = 9).

Infants with visible eczema were significantly more likely to have enrollment IgE levels exceeding the 0.1 kU/L threshold (no eczema, 10.0%; visible eczema, 32.2%; *P* < .001). Furthermore, among those with visible eczema, the likelihood of exceeding the 0.1 kU/L threshold showed a dose response with SCORAD severity: mild (SCORAD <15) 22.4%, moderate (SCORAD 15 to <40) 60.0%, and severe (SCORAD ≥40) 88.9% (*P* < .001).

### Ethnicity

One thousand one hundred four (84.7%) of the enrolled infants were white, and 199 were nonwhite (15.3%). The nonwhite group consisted of 119 infants of mixed ethnicity (9.1%) and 80 (6.1%) black, Asian, or Chinese infants. Enrollment sensitization was very strongly associated with ethnicity. Sensitization at enrollment to 1 or more foods exceeding the 0.1 kU/L threshold was present in 12.3% of white participants, 22.9% participants of mixed ethnicity, and 48.6% of black, Asian, or Chinese participants (*P* < .001). Enrollment sensitization based on skin prick test responses in the EIG was also strongly associated with ethnicity: 3.6% of white participants had positive skin prick test responses to 1 or more foods (wheal of any), as did 4.2% of participants of mixed ethnicity and 23.4% of participants of black, Asian, or Chinese ethnicity (*P* < .001).

### Sensitization

Skin prick tests were undertaken in all 652 EIG infants (Table I). Specific IgE levels were measured successfully in 1170 participants (SIG, 89% [577/651]; EIG, 91% [593/652]). Enrollment sensitization was more frequent when defined by specific IgE results (Table I). Of EIG participants, 5.1% were sensitized to 1 or more foods on skin prick tests (>0 mm wheal) compared with 5.7% using a specific IgE threshold of 0.35 kU/L or greater and 15.7% using a threshold of 0.1 kU/L or greater. There were no significant baseline differences between either group on specific IgE testing at either threshold level for any food or for individual foods (Table I).

**Table I:** Enrollment SPT and specific IgE sensitization data from the EAT study

	Peanut	Egg	Milk	Sesame	Fish	Wheat	Any food
SPT >0 mm							
EIG	1.2% (8/652)	3.7% (24/652)	1.5% (10/652)	0% (0/652)	0% (0/652)	0.2% (1/652)	5.1% (33/652)
Specific IgE (kU/L)							
Mean	0.07	0.20	0.11	0.06	0.004	0.07	
Median	0.03	0.01	0.02	0.06	0.00	0.04	
Range	0-11.60	0-42.22	0-57.49	0-0.33	0-0.09	0-12.91	
Specific IgE ≥0.1 kU/L							
All participants	3.6% (42/1166)	6.7% (78/1170)	6.0% (70/1169)	2.0% (23/1151)	0% (0/1164)	4.3% (50/1165)	15.6% (182/1170)
SIG	3.1% (18/576)	7.3% (42/577)	6.6% (38/576)	1.4% (8/572)	0% (0/575)	4.3% (25/576)	15.4% (89/577)
EIG	4.1% (24/590)	6.1% (36/593)	5.4% (32/593)	2.6% (15/579)	0% (0/589)	4.2% (25/589)	15.7% (93/593)
Specific IgE ≥0.35 kU/L							
All participants	1.6% (19/1166)	3.9% (45/1170)	2.8% (33/1169)	0% (0/1151)	0% (0/1164)	0.9% (10/1165)	6.4% (74/1170)
SIG	1.6% (9/576)	4.7% (27/577)	3.3% (19/576)	0% (0/572)	0% (0/575)	1.0% (6/589)	6.9% (40/577)
EIG	1.7% (10/590)	3.0% (18/593)	2.4% (14/593)	0% (0/579)	0% (0/589)	0.7% (4/576)	5.7% (34/593)

Specific IgE levels were measured in 1170 children. However, some infants had very small amounts of serum obtained, and levels for all 6 individual foods could not be measured. Hence the denominator for individual foods varies (ranging from n = 1151 for sesame to n = 1170 for egg).

Sensitization rates varied significantly by food in both forms of testing. Egg results were most commonly positive, whereas in contrast, not 1 infant was sensitized to fish based on specific IgE levels or skin prick test responses. Milk sensitization was more common than peanut sensitization on both skin prick testing and specific IgE measurements.

### Adherence to the early introduction regimen among groups at high risk of food allergy

In univariate analyses EIG participants with eczema were less likely to adhere to the early introduction regimen (no eczema, 44.7%; visible eczema, 34.4%; *P* = .03). There was also a trend toward greater rates of nonadherence being associated with more significant eczema at enrollment (44.7% adherent with no eczema, 37.4% with mild SCORAD, 29.6% with moderate SCORAD, and 0% with severe SCORAD; *P* = .01, test for trend). However, the strongest univariate association with EIG nonadherence was shown with ethnicity: white, 44.3% (206/465); mixed, 32.4% (12/37); and Asian/black/Chinese, 13.3% (4/30; *P* < .001). Adherence to the early introduction regimen in the EIG did not differ significantly by enrollment sensitization status using a threshold of 0.1 kU/L or greater: 37.8% of sensitized EIG participants were adherent compared with 43.5% of nonsensitized EIG participants (*P* = .36).

When eczema at enrollment, ethnicity, and sensitization at enrollment (whether determined based on results of skin prick testing or specific IgE measurement) were included in a logistical model with EIG adherence as the outcome, only ethnicity remained significant (data not shown).

### Efficacy of the early introduction regimen among groups at high risk of food allergy

#### Enrollment eczema

In an intention-to-treat analysis of infants with any visible eczema at the enrollment visit, there were reductions in the EIG for allergy to 1 or more foods (SIG, 18.2%; EIG, 15.6%; *P* = .56), egg allergy (SIG, 14.0%; EIG, 9.8%; *P* = .27), and peanut allergy (SIG, 7.0%; EIG, 2.8%; *P* = .10), with the latter approaching statistical significance.

**Fig 1:**
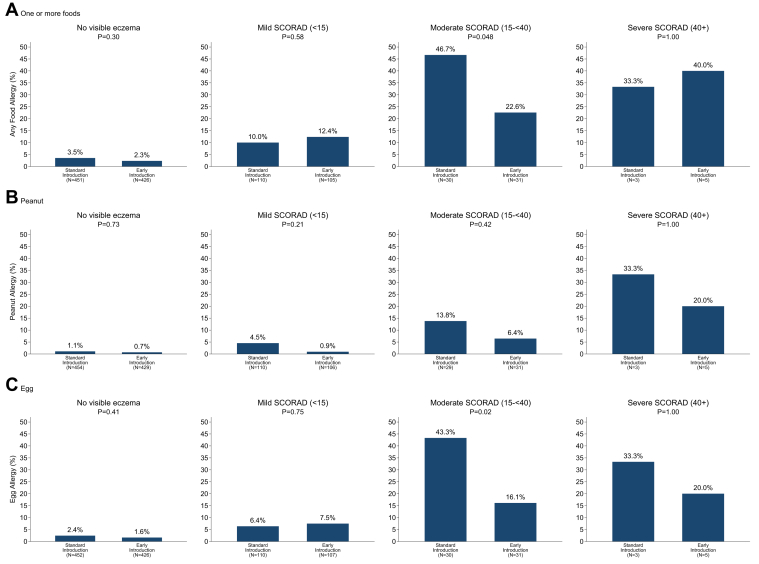
Intention-to-treat efficacy of the EAT study by enrollment eczema SCORAD score group.

Analysis of intention-to-treat efficacy among infants with visible eczema categorized by grouped eczema severity status (SCORAD), see Fig 1, showed efficacy in the EIG infants with moderate SCORAD eczema for the outcome of allergy to 1 or more foods (SIG, 46.7%; EIG, 22.6%; *P* = .048) and for egg allergy (SIG, 43.3%; EIG, 16.1%; *P* = .02). There was also a 79% reduction in peanut allergy in the mild SCORAD group (SIG, 4.5%; EIG, 0.9%), but it was not statistically significant (*P* = .21). Results of formal statistical interaction tests between study group and grouped SCORAD were not significant.

However, when analyzing SCORAD as a continuous variable, there was a statistically significant interaction effect for the association between study group and continuous SCORAD for egg allergy (interaction term OR, 0.95; *P* = .028).

There were too few participants with severe SCORAD eczema for statistical comparisons between the 2 study groups to be robust. If the groups with moderate and severe SCORAD were combined, the risk reduction was similar to that of the moderate SCORAD group alone, and the significance test remained statistically significant for egg (*P* = .02) but was of borderline significance for the primary outcome (*P* = .07).

#### Ethnicity

**Fig 2:**
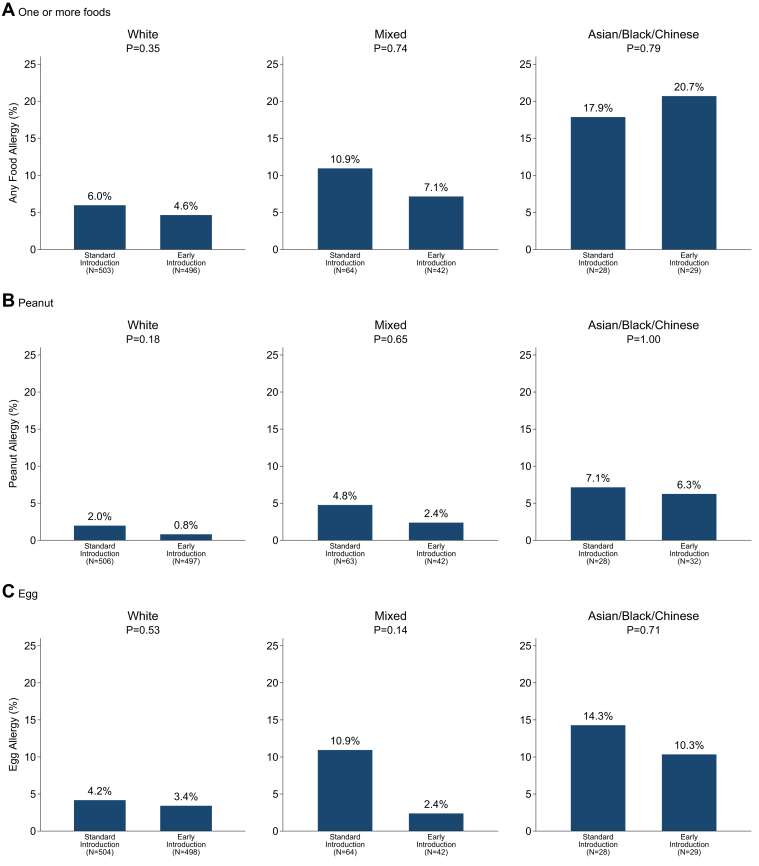
Intention-to-treat efficacy of the EAT study by ethnicity.

Despite the very low rates of adherence in the Asian, black, or Chinese infants in the EIG, rates of food allergy in the EIG were lower in 8 of the 9 comparisons within ethnic groups in Fig 2. However, there were no statistically significant intention-to-treat effects for any of the food allergy outcomes.

In contrast, allergy to 1 or more foods showed a stepwise increase from white participants (both groups combined, 5.3%) to mixed ethnicity (9.4%), with the highest prevalence being observed in Asian, black, or Chinese participants (19.3%, *P* < .0005). The same pattern is seen in Fig 2 within the SIG (*P* = .03) and the EIG (*P* = .001) separately.

#### Infants sensitized at enrollment

**Fig 3:**
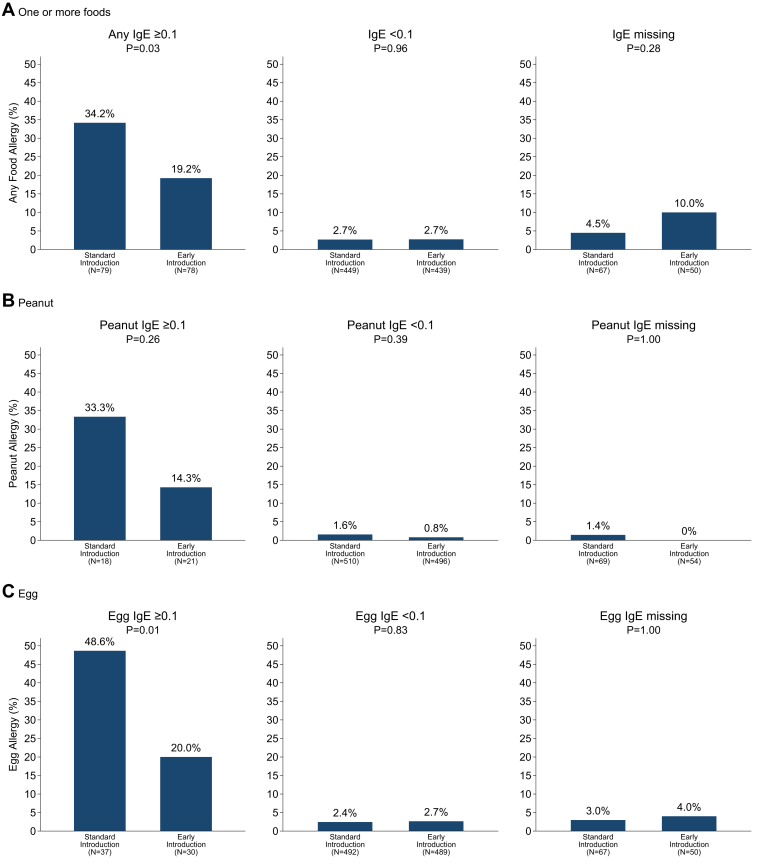
Intention-to-treat efficacy of the EAT study among infants sensitized (IgE ≥0.1 kU/L) at enrollment.

For more information on infants sensitized (≥0.1 kU/L) at enrollment, see Fig 3. The primary outcome, allergy to 1 or more foods, could be determined in 89% (1045/1170) of participants with enrollment IgE sensitization data to 1 or more foods. Peanut allergy status could be determined in 89% (1041/1166) of those with enrollment peanut IgE sensitization data and egg allergy status in 89% (1045/1170) of those with enrollment egg IgE sensitization data.

Of the 157 infants who were sensitized to 1 or more foods (≥0.1 kU/L) whose primary outcome status was evaluable, 34.2% (27/79) of SIG participants developed food allergy to 1 or more foods versus 19.2% (15/78) of EIG participants (*P* = .03). Thirty-nine infants were sensitized to peanut at 0.1 kU/L or greater, and the corresponding figures for developing a peanut allergy in infants whose peanut allergy status was evaluable were as follows: 33.3% (6/18) for SIG infants versus 14.3% (3/21) for EIG infants (*P* = .26). Sixty-seven infants were sensitized to egg at 0.1 kU/L or greater; egg allergy developed in those whose egg allergy status was evaluable in 48.6% (18/37) of SIG infants versus 20.0% (6/30) of EIG infants (*P* = .01). Adjusting for age, ethnicity, sex, and maternal age did not affect the results.

A statistical interaction test between study group and sensitization was significant for egg (interaction term *P* = .041) but not for peanut (*P* = .673) or sensitization to 1 or more foods (*P* = .149).

Among infants with no enrollment sensitization to any food or specifically to peanut or egg, there were no significant differences in food allergy rates between the 2 groups. Similarly, among those without enrollment IgE data, there were no significant differences between the 2 groups in the intention-to-treat analysis.

No sample was available to measure specific IgE levels in 133 participants at 3 months of age (74 SIG and 59 EIG infants). Being in this group was not associated with ethnic status, visible eczema at enrollment, or the presence of sensitization on skin prick tests. A sensitivity analysis was undertaken, apportioning those infants with IgE data missing to the sensitized or nonsensitized groups in accordance with the prevalence of sensitization in those with data. The efficacy of the intervention for egg persisted, and the formal interaction test remained statistically significant.

There are no existing data on allergen-specific IgE levels or skin prick test responses as predictors of challenge-proved food allergy in 3-month-old infants. Only 7 of the 652 EIG infants had positive enrollment food challenge results (milk, 4; egg, 2; peanut, 2; and wheat, 1). These numbers are too small to generate positive predictive values in this age group.

## Discussion

A recent systematic review of all published early intervention trials undertaken to date concluded that early egg or peanut introduction to the infant diet was associated with lower risk of developing egg or peanut allergy.^12^

The LEAP study showed a very strong intention-to-treat effect. However, this was in the context of an extremely high per-protocol adherence rate (96%), which was achieved through regular sustained contact with the families. The EAT study intervention was considerably more challenging, with multiple foods being introduced at a young age. This, combined with a more pragmatic contacting regimen, had a concomitant effect of significantly reducing the per-protocol adherence rate.

In this article we have shown that despite significantly lower adherence rates than in the LEAP study, groups of infants at high risk of developing a food allergy, including those sensitized at enrollment and those with increasing eczema severity (SCORAD) benefited from early introduction of allergenic solids. Of equal importance was that early introduction of allergenic foods into the diets of the non–high-risk infants was not associated with any increased risk of food allergy.

It is important to note that we investigated the efficacy of the EAT intervention in these groups of infants because they are known to be at particularly high risk of developing a food allergy. Not all of these groups are readily identifiable: infants with nonwhite ethnicity and those with visible eczema are, and hence could benefit from targeted support with recommendations for early food introduction. Conversely, although we are not proposing IgE screening in the general population from which the EAT study population was recruited, it is of significance that EAT study early food intervention is being shown to have been effective in an intention-to-treat analysis in this group of infants.

Current National Institute of Allergy and Infectious Diseases guidelines propose introduction of peanut from around 6 months of age in children with mild-to-moderate eczema.^13^ In the guidelines the authors state that their recommendation is based on 2 pieces of evidence: first, evidence from the LEAP study, in that some infants who participated in the LEAP study based on an egg allergy had a SCORAD at screening in the mild-to-moderate category, and second, the per-protocol protective effect seen with early peanut introduction in the EAT study.^13^ Of interest is our identification of a significant intention-to-treat effect in children with moderate eczema (52% reduction in allergy to ≥1 foods and 63% reduction in egg allergy) and with increasing eczema SCORAD for egg allergy. This adds to the evidence presented in these guidelines.

The principal limitation of these analyses is that only the analysis of the efficacy of the early introduction intervention in infants with eczema at enrollment was stipulated *a priori*. Investigating the effects of the intervention in sensitized infants and nonwhite infants are *post hoc* subgroup analyses that are subject to well-recognized limitations. If important subgroup effects are anticipated, trials should either be powered to detect them reliably, or pooled analyses of several trials should be undertaken.^14^

A further limitation is the low adherence rate in the EAT study, reducing the power to detect an ITT intervention effect in the EAT study. We have explored in detail which factors are associated with low adherence in the EAT cohort. In that publication we modelled the effect of improving adherence in the groups at high risk of developing a food allergy (nonwhite participants and those with early-onset eczema) and showed that this has the potential to significantly reduce the burden of food allergy if sufficiently high adherence were able to be achieved.^15^

The EAT study intervention did not show intention-to-treat efficacy when children with visible eczema were considered as one group, but efficacy was present within the moderate SCORAD subgroup and with continuous SCORAD for egg allergy. The number of infants with severe SCORAD eczema in the EAT study was too small to draw any conclusions about the efficacy of the intervention within this subgroup within the EAT study. We discuss the reasons for this in the Discussion section in this article's Online Repository at www.jacionline.org.

In the LEAP study, peanut-specific IgE levels of 0.1 kU/L or greater in infancy were clinically and statistically significant in terms of predicting peanut allergy at 60 months of age. Overall, 75% (48/64) of cases of peanut allergy in the LEAP study came from those with peanut-specific IgE levels of 0.1 kU/L or greater at baseline, and in the avoidance group this number was 74% (40/54). In LEAP participants who had egg allergy with mild or no eczema at enrollment, the proportion developing peanut allergy, with IgE levels to peanut of 0.1 kU/L or greater at baseline, was 89% (8/9) overall and 88% (7/8) in the avoidance group (the *P* value for this subgroup analysis within the mild eczema group [n = 105] was significant: *P* = .0002). The clinical significance of this threshold was confirmed in the EAT study, with 69% of those developing a food allergy in the SIG by 3 years of age already having specific IgE present to 1 or more of the early introduction foods at the 0.1 kU/L threshold at 3 months of age.

As countries, including the United States,^16^ Australia,^17^ and the United Kingdom,^18^, ^19^ move to issue new infant feeding guidelines in light of EAT and LEAP study findings, as well as the other randomized trials that have taken place of early food introduction, we hope our findings will inform the debate as to whether a risk-based dietary intervention should be recommended or a population-based intervention should be undertaken.

**Clinical implications**

The EAT study was effective in certain groups of infants at high risk of developing food allergy in an intention-to-treat analysis, with significant implications for new infant feeding recommendations.

## Discussion

The LEAP study has clearly shown that infants with severe eczema benefit significantly from early peanut introduction.^E1^ In the EAT study protocol, *a priori*, we predicted that 25% of infants would have visible eczema at the 3-month assessment, and we estimated the prevalence of food allergy in these infants to be 30%.^E2^ By using this estimate, the study had 99% power to show a reduction from 30% to 10% in food allergy and 85% power to detect a reduction from 30% in the SIG to 15% in the EIG.^E2^ However, although the estimate of the proportion of infants with any visible eczema at enrollment was accurate (24.4%), the rate of any food allergy in the SIG infants with visible eczema at enrollment was significantly lower than anticipated (18.2% as opposed to 30%). This reflects the preponderance of mild eczema among those with visible eczema at enrollment and meant that the study was potentially underpowered to explore eczema subgroup efficacy. The fact that we found significant findings despite this is noteworthy. Furthermore, we are now undertaking a case-based pooled analysis of several of the early food introduction trials to optimally investigate the efficacy of early food introduction in these subgroups.^E3^
